# Exploring cariprazine as a treatment option for varied depression symptom clusters

**DOI:** 10.3389/fpsyt.2024.1442699

**Published:** 2024-09-25

**Authors:** Bojana Pejušković, Ana Munjiza Jovanović, Danilo Pešić

**Affiliations:** ^1^ Clinical Department for Crisis Intervention and Affective Disorders, Head, Institute of Mental Health, Belgrade, Serbia; ^2^ School of Medicine, University of Belgrade, Belgrade, Serbia; ^3^ Clinic for Children and Adolescence, Institute of Mental Health, Belgrade, Serbia

**Keywords:** treatment-resistant major depressive disorder, depression symptom clusters, atypical antipsychotics, D2/D3 partial agonists, cariprazine

## Abstract

Major depressive disorder (MDD) is among the most prevalent psychiatric conditions and a leading cause of disability worldwide. MDD presents a diverse range of symptoms that significantly impact personal, societal, and economic dimensions. Despite the availability of numerous antidepressant treatments (ADTs) targeting different molecular mechanisms, a substantial proportion of patients experience inadequate response, presenting a considerable challenge in MDD management. As a result, adjunctive strategies, particularly involving atypical antipsychotics, are often employed to enhance treatment efficacy. Cariprazine, a D2/D3 partial agonist, is distinguished from other atypical antipsychotics by its selective action on the D3 receptor and its modulation of 5-HT1A, 5-HT2A, and alpha 1B receptors. This distinctive pharmacological profile warrants investigation into its potential effectiveness and tolerability across various symptom domains of MDD, including pleasure, interest, and motivation; mood and suicidality; sleep and appetite; fatigue; psychomotor activity and anxiety; and cognitive function. Preliminary evidence from animal studies and clinical trials suggests that cariprazine may improve motivation, anhedonia, and cognitive function symptoms. Cariprazine shows promise in alleviating mood-related symptoms, though its impact on anxiety and its effects on agitation and psychomotor retardation remains uncertain. Cariprazine may be particularly beneficial for patients with MDD exhibiting anhedonia, cognitive deficits, and possibly fatigue and hypersomnia. Evaluating cariprazine’s efficacy across these symptom domains could reveal patterns that support more personalized treatment approaches for depression. Further research is essential to elucidate the role of cariprazine as an adjunctive therapy for adults with major depressive disorder who have an inadequate response to antidepressant monotherapy.

## Introduction

Major depressive disorder (MDD) ranks among the most prevalent psychiatric disorders and stands as a leading cause of disability worldwide (1, 2). It affects an estimated 246-286 million individuals globally (3). Approximately 9-26% of females and 5-12% of males will experience at least one episode of MDD during their lifetime, with around 50% of these individuals likely to experience recurrent episodes (4–6).

Major depressive disorder encompasses a broad spectrum of symptomatology, impacting personal, societal, and economic domains (7, 8). It is linked to a diminished quality of life, elevated rates of suicidal behavior, and general mortality (9). The associated disability leads to increased absenteeism, resulting in substantial productivity losses and imposing a significant burden on healthcare and economic systems. Furthermore, MDD is projected to become one of the three leading causes of disease burden worldwide by 2030 (10). The impact is further compounded by the recognition of depression as a major risk factor for other mental disorders, particularly substance use disorders, post-traumatic stress disorder, and anxiety disorders (11, 12). Additionally, depression has been identified as an independent risk factor and negative prognostic indicator for numerous chronic somatic disorders, including diabetes, cardiovascular disease, hypertension, chronic respiratory disorders, arthritis, and cancer (13–15).

Depression is a heterogeneous entity characterized by a variety of complex symptoms. In the DSM-5 and ICD-11 classifications, the symptoms listed for the diagnosis of depression are grouped into nine largely identical categories, although hopelessness about the future is specifically mentioned only in the ICD-11 (16, 17). In both systems, the presence of at least five of these symptoms most of the day, nearly every day, for at least two weeks is required, with the occurrence of either depressed mood or diminished interest in pleasurable activities being mandatory. The nine symptom groups include: depressed mood; markedly diminished interest or pleasure in activities; reduced ability to think or concentrate, or indecisiveness; feelings of worthlessness, or excessive or inappropriate guilt; recurrent thoughts of death, suicidal ideation, or suicide attempts or plans; insomnia or hypersomnia; a significant change in appetite or weight; psychomotor agitation or retardation; and fatigue or loss of energy (18).

**Figure 1:**
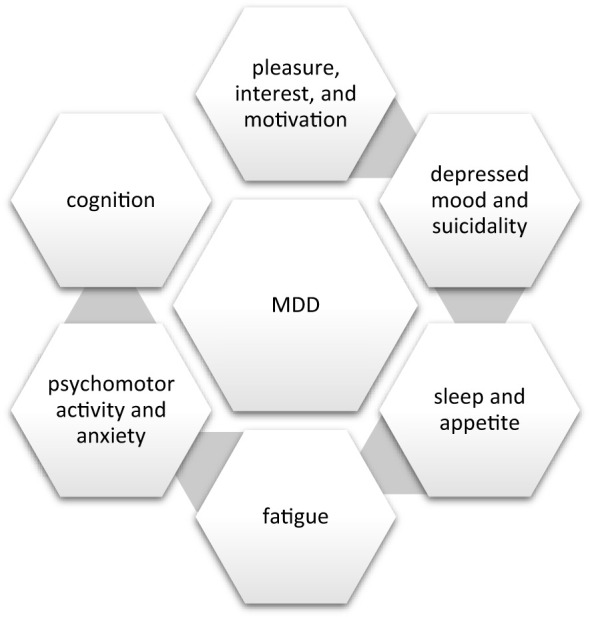
Principal domains of major depressive disorder.

Steven Stahl, a leading authority in psychopharmacology, provided a significant framework for understanding depression by defining it as a syndrome and describing five key dimensions of symptoms: vegetative, cognitive, impulse control, behavioral, and physical (somatic) (19). His comprehensive approach has greatly enhanced the understanding and treatment of depression by emphasizing its multifaceted nature. A recent study utilizing a national telehealth sample on the symptom clustering of major depression identified five components: anxious distress, core emotions, agitation/irritability, insomnia, and anergic/apathy (20). Despite these categorizations, depression remains a complex and heterogeneous syndrome with varying symptom clusters. Our symptom clusters within MDD are presented in **Figure 1**.

The primary treatment for depression, according to various guidelines, is pharmacotherapy, with antidepressants as the first line of treatment, often complemented by psychotherapy or a combination of both (21). Despite the availability of a wide range of antidepressant treatments (ADT) targeting different molecular mechanisms, insufficient treatment response remains a significant challenge (22). Studies indicate that only approximately half of patients exhibit a favorable response to initial ADT, while only one-third experience remission. The remaining two-thirds often experience an inadequate response to one or more ADTs of sufficient dose and duration of treatment (23).

Current recommendations for patients who do not respond to initial antidepressant therapy (ADT) include switching to another antidepressant within the same class or to a different class, or employing a combination of antidepressants with different mechanisms of action (21, 24). Additionally, adjunctive strategies are frequently utilized in clinical practice, with the addition of atypical antipsychotics being the most common approach (25). Other adjunctive options include the use of lithium, thyroid hormones, dopamine compounds, ketamine and non-pharmacological treatments (24).

Data indicates that antipsychotics are widely used as adjunctive therapy for MDD in the United States, with approximately 3.9 million treatment visits annually in 2007 and 2008 involving antipsychotic prescriptions, predominantly second-generation antipsychotics (25). Currently, five atypical antipsychotics—aripiprazole, quetiapine XR, olanzapine, brexpiprazole, and cariprazine—are approved by the FDA for adjunctive treatment of adult treatment-resistant depression (TRD) (26).

### Cariprazine

Cariprazine, an orally active antipsychotic and dopamine partial agonist, has emerged as a significant advancement in the treatment of psychiatric disorders. Although the precise mechanism of its antidepressant activity remains unknown, its pharmacological profile likely contributes to its efficacy. Cariprazine acts as a dopamine antagonist/agonist, with actions ranging from “silent” antagonism to “full” agonism, the maximum stimulation of the D2 receptor (27). Its selective effects on the D3 receptor and the 5HT1A, 5HT2A, and alpha 1B receptors distinguish it from other atypical antipsychotics. This unique mechanism has demonstrated efficacy across a broad spectrum of schizophrenia symptoms, particularly negative and cognitive symptoms, making it a “drug of choice” for these clinical dimensions (28, 29). Furthermore, cariprazine is highly effective in treating bipolar disorder, representing a state-of-the-art treatment for both bipolar depression and mania, according to Mazza and collaborators (30). Due to its proven efficacy and safety, cariprazine was initially FDA-approved for the treatment of schizophrenia and bipolar I disorder. In December 2022, it received approval as an augmentative therapy for MDD. The recommended doses for cariprazine vary depending on the condition: 1.5–6 mg/day for schizophrenia, 3–6 mg/day for acute manic/mixed episodes, 1.5–3 mg/day for depressive episodes associated with bipolar disorder, and 1.5 or 3 mg/day as an adjunctive therapy for MDD (31).

## Objectives and methodology

We aimed to investigate the potential effectiveness and tolerability of cariprazine augmentation treatment across various domains of MDD. These domains include pleasure, interest, and motivation; depressed mood and suicidality; sleep and appetite; fatigue; psychomotor activity and anxiety; and cognitive function.

The information utilized to compose this manuscript was meticulously collected from the sources listed in **Table 1**. This comprehensive review aims to provide a detailed understanding of cariprazine’s impact on these critical aspects of MDD, contributing to the broader knowledge base and offering insights for clinical practice.

**Table 1:** Sources used for this review.

**1.**	Medline search from January 2000 to January 2024. Keywords: Major depressive disorder (MDD), cariprazine, depression symptom clusters, treatment-resistant MDD, D2/D3 partial agonists, and atypical antipsychotics. Types of articles: original articles, clinical trial, meta-analysis, randomized controlled trial, review, systematic review
**2.**	Cochrane Library database search from January 2000 to January 2024. Keywords: Major depressive disorder (MDD), cariprazine, depression symptom clusters, treatment-resistant MDD, D2/D3 partial agonists, and atypical antipsychotics.
**3.**	Hand search of references of retrieved literature
**4.**	Personal and college libraries searching for texts on research methods and literature reviews.
**5.**	Discussions with experts in the field of reviews of the literature
**6.**	Personal experience participating in and writing several reviews of the literature

## Results

A substantial number of sources were identified during the literature search dedicated to investigating the effects of cariprazine on the core dimensions of MDD. These sources include a variety of preclinical studies, clinical trials, randomized controlled trials, as well as extensive reviews and systematic reviews. **Table 2** lists the double-blind controlled studies in which cariprazine was used in the treatment of MDD.

**Table 2:** List of double-blind, randomized, placebo-controlled studies that used cariprazine for MDD treatment.

No.	Clinical trial	Cariprazine dosage (N)	Duration of treatment	Study design	Measures
**1.**	Vieta E. et al., 2019 (32)	flexible-dose (1.5–4.5 mg/day) (N=311)	long-term (26-week)	Double-blind: placebo + ADT (n=109), cariprazine +ADT (n=108), single-blind placebo + ADT (n=94)	Montgomery–Åsberg Depression Rating Scale; Clinical Global Impression-Severity score; Columbia-Suicide Severity Rating Scale; Barnes Akathisia Rating Scale; Simpson-Angus Scale; Abnormal Involuntary Movement Scale
**2.**	Fava M. et al., 2018. (33)	cariprazine at 0.1–0.3mg/day or 1.0–2.0 mg/day (N=230)	8-weeks open-label + 2-week safety follow-up	Double-blind: ADT + placebo group (n=81); ADT + cariprazine 0.1–0.3 group (n=76); ADT + cariprazine 1–2 mg/day group (n=73)	Montgomery–Åsberg Depression Rating Scale; Clinical Global Impression-Severity score; Hamilton Depression Rating Scale
**3.**	Earley W. R. et al., 2018 (34)	flexible-dose (1.5–4.5mg/day) (N=525)	8-weeks open-label + 1-week safety follow-up	Double-blind: ADT + placebo group (n=258); ADT + cariprazine group (n=267)	Montgomery–Åsberg Depression Rating Scale; Clinical Global Impression-Severity score; Sheehan Disability Scale; Hamilton Rating Scale for Anxiety; Hamilton Depression Rating Scale
**4.**	Riesenberg R. et al., 2023 (35)	cariprazine at 1.5mg/day or 3mg/day groups (N=501)	6 weeks treatment period + 4-week safety follow-up	Double-blind: ADT + placebo group (n=249); ADT + cariprazine 1.5mg/day group (n=250); ADT + cariprazine 3mg/day group (n=251)	Montgomery–Åsberg Depression Rating Scale; Clinical Global Impression-Severity score; Hamilton Rating Scale for Anxiety; Hamilton Depression Rating Scale
**5.**	Durgam S. et al., 2016. (36)	cariprazine at 1-2mg/day or 2-4.5mg/day (N=550)	8 weeks treatment period + 1-week safety follow up	Double-blind: ADT + placebo group (n=269); ADT + cariprazine 1-2mg/day group (n=274); ADT + cariprazine 2-4.5mg/day group (n=276)	Montgomery–Åsberg Depression Rating Scale; Columbia-Suicide Severity Rating Scale; Clinical Global Impression-Severity score; Sheehan Disability Scale
**6.**	Sachs S.G. et al., 2023. (37)	cariprazine at 1.5 mg/day, or at 3.0 mg/day (N=.502)	6 weeks treatment period + 4-weeks safety follow-up	Double-blind: ADT + placebo group (n=249); ADT + cariprazine 1.5mg/day group (n=250); ADT + cariprazine 3mg/day group (n=252)	Montgomery–Åsberg Depression Rating Scale; Hamilton Depression Rating Scale; Hamilton Rating Scale for Anxiety; Clinical Global Impressions severity

### Pleasure, interests, motivation

Some of the most prominent symptoms of depression are loss of motivation, interest, and/or pleasure in activities a person previously felt satisfaction. From neurobiological perspectives, regions of the brain that are included in the pathogenesis of these symptoms by alteration in neural activity are the ventral tegmental area (VTA), ventral pallidum, the striatum (especially the nucleus accumbens-NAc), amygdala, hippocampus, anterior cingulate, insula, orbitofrontal cortex and the ventromedial prefrontal cortex (VMPFC) (38–40). Dopaminergic transmission modulates several important characteristics and dimensions of reward, including anticipation, motivation, effort, and learning (41).

Dysregulation of the dopamine system has been observed in both bipolar disorder and MDD, with some findings suggesting reduced dopaminergic activity, specifically in unipolar depression (42–44). This dysregulation may play a significant role in the pathophysiology of depressive symptoms, particularly impacting motivation and subsequently affecting cognitive function and mood.

Cariprazine, a potent dopamine D2 and D3 partial agonist, exhibits a significantly higher affinity for D3 receptors than D2 receptors (27). These D3 receptors are predominantly expressed in the limbic system, particularly in regions associated with motivation and reward-related behaviors (45). The nucleus accumbens shell, a critical brain region, exhibits increased D3 receptor expression following cariprazine treatment. This upregulation is proposed as a common neurobiological mechanism underlying the antidepressant efficacy (46). Distinct from other antipsychotics, cariprazine binds strongly to D3 receptors and increases dopamine D3 receptor levels in D3 receptor-rich brain regions with chronic treatment (47).

This data is supported by research in animal models, where cariprazine has shown positive effects on effort-based choice behavior, an animal model of avolition (48). The first promising results and indications that cariprazine will improve loss of motivation and impaired reward behavior in depressed patients are present for 10 years (49), due to fact that reduced activation of the mesolimbic dopaminergic pathway is involved in the loss of motivation and/or anhedonia (50) and that these areas are with highly expressed D3 receptors – targets for cariprazine. The results in animal model studies have shown that stimulation of D2/D3 receptors in the nucleus accumbens with D2/D3 agonists induces an effort-related decision-making pathway, which is included in the reword association process (51). Another study demonstrated that cariprazine attenuated anhedonia in the animal chronic mild stress model of depression (52). Data on cariprazine’s effects on impaired motivation and anhedonia in individuals with MDD are not yet consistent. However, some promising results have been observed in MDD case studies and among patients with anhedonia within bipolar disorder (53).

### Mood and suicidality

Numerous brain regions are involved in the pathogenesis of depressed mood, including the prefrontal cortex, hippocampus, temporal lobe, thalamus, striatum, and amygdala (54). Key areas within the frontal lobe include the anterior cingulate cortex (ACC), orbitofrontal cortex (OFC), and middle prefrontal cortex (54–57). Numerous neural network structures are involved in mood regulation, while endocrine and immune factors, along with neurotransmitters, play significant roles in the biochemical pathophysiology of mood-related symptoms of depression. The three principal neurotransmitters associated with the genesis of depressed mood and its treatment are dopamine, serotonin, and norepinephrine (58). All of them are widely distributed in all brain regions mentioned above. Research on animal models showed that cariprazine behaves as a 5-HT1A autoreceptor agonist in the dorsal raphe nucleus, as a 5-HT2A receptor antagonist in modulating the firing activity of locus coeruleus norepinephrine neurons, and as a full agonist at 5-HT1A receptors mediate the electrophysiological effect of 5-HT on pyramidal neurons (59). Also, animal model research found an increase in neurotransmission at 5-HT1A receptors in cariprazine-treated rats in the hippocampus and increase locus coeruleus noradrenergic neuron activity after 14 days of cariprazine administration (60). We can hypothesize that this is the mechanism by which cariprazine could affect mood-related symptoms of depression.

The results of studies on the effects of cariprazine on depression have been mixed. Nonetheless, a considerable number of research studies and meta-analyses suggest that adding cariprazine as an augmentation therapy significantly alleviates depressive symptoms compared to placebo (24, 36, 61). On the other side, some of it failed to prove the efficacy of cariprazine augmentation in patients with MDD with inadequate previous response to antidepressants (34, 35). The majority of double- blind randomized controlled trials that explored cariprazine as an augmentation therapy to an antidepressant in the treatment of major depressive disorder or therapy-resistant depression showed that it is not efficient or it is less efficient in dozing under 2-4.5mg or 3mg per day (33, 35, 36). On the other hand, a few authors found that a lower dose of cariprazine, under 3mg/day, was efficient in the reduction of depressive symptoms in adults with major depressive disorder compared to a higher dose of 3mg/day (37). One study showed that more than 53% of patients with MDD who were treated with antidepressant and cariprazine augmentation in flexible doses (range 1.5-4.5 mg/d after week 26 were in remission by MADRS scale. Still, the differences between low and high cariprazine dosage were not analyzed (32). There is also the matter of several studies that observed a non-significant reduction in depressive symptoms without analyzing the doses of cariprazine, which varied flexibly within the range of 1.5-4.5 mg/day (34). All these facts indicate cariprazine efficiency in mood-related symptoms of depression, but there is no right dose that is unique for all phenotypes of depression and without consideration of other metabolic and pharmacodynamic characteristics of a person who suffers from it. In addition to this theory, in the animal chronic mid-stress model, cariprazine attenuated anhedonia symptoms (represented as reductions in sucrose intake) in a wide range of dosages but not in the lowest and highest dose (52).

There is no specific research regarding cariprazine’s effects on suicidality in depressed patients. Still, the majority of publications report low rates of suicidal ideation in patients with MDD treated with cariprazine in the range between 5-8% (34, 35, 61). On the other side, some authors emphasize there was no reported suicidal behavior or the appearance of suicidal ideation in patients during cariprazine treatment (33, 36). In research by Vieta et al., out of 442 patients who were included in cariprazine treatment as adjuvant therapy for MDD, one patient experienced suicidal ideations that were treatment-related (32). Regarding suicidality, Riesenberg and his colleagues reported in their research that the rates were 5.2% for a dose of 1.5 mg/day and 7.6% for a dose of 3 mg/day, compared to 6.4% in the placebo group. It is important to emphasize that these results were obtained from a study involving 501 patients taking cariprazine and 250 placebo controls. Notably, most events were in the least severe category (“a wish to be dead”), and none of the patients exhibited suicidal behavior or committed suicide (35). In the study of Earley (34) et al. also no suicidal behavior was reported, and suicidal ideation was present in the already mentioned range (around 8%). Suicidal behavior was not reported in 149 Patients with MDD taking cariprazine and AD during double-blind treatment in a study of Fava and colleagues, and the incidence of suicidal ideation was higher in patients treated with placebo than either cariprazine dose as measured with the Columbia-Suicide Severity Rating Scale (19.8%, placebo; 11.8%, cariprazine 0.1–0.3 mg/day; and 12.3%, cariprazine 1.0–2.0 mg/day) (33). Understanding the mechanisms of suicidality in MDD is of paramount importance in the field of psychiatry. Despite extensive research, the precise mechanisms by which certain psychotropic drugs mitigate suicidal behavior remain unclear. Available evidence indicates that cariprazine either reduces suicidality or, at the very least, does not exacerbate it in patients with depression.

### Sleep and appetite

Depression as a syndrome encompasses various symptoms, including changes in sleep and appetite. Patients may experience hypersomnia, difficulty falling asleep, waking up during the night, early morning awakenings, or fluctuating appetite—from loss of appetite to increased appetite. Given these diverse symptoms, expecting a single drug to address all variations arising from alterations in neurotransmitters, hormones, and neural circuits involved in regulating sleep and appetite is challenging.

Circuits in the hypothalamus regulate sleep and wakefulness continuity. In a simplified model, the lateral hypothalamus, which stabilizes and promotes wakefulness, is balanced by melatonin-sensitive neurons in the suprachiasmatic nucleus, the brain’s internal clock. Two key neurotransmitters involved in the sleep/wake switch are histamine from the tuberomammillary nucleus and γ-aminobutyric acid (GABA) from the ventrolateral preoptic nucleus. Additionally, orexin-containing neurons in the lateral hypothalamus and melatonin-sensitive neurons in the suprachiasmatic nucleus regulate sleep/wake homeostasis in opposite ways, with melatonin promoting sleep and orexin promoting nighttime arousal (62, 63). It’s important to note that 5HT2A/2C agonists also promote wakefulness. Consequently, antagonists of 5HT2A/2C receptors are being utilized as sleep-promoting drugs (64).

Cariprazine exhibits activity as a partial agonist at serotonin (5-HT) receptors, including 5-HT2A and 5-HT2C receptors, while demonstrating modest antagonist activity at histamine (H) receptors. Although cariprazine acts as an H1 antagonist, it is only a moderate one, which may result in sedative effects. Despite this, cariprazine can also cause insomnia due to its complex pharmacological profile, including actions on dopamine D2 and D3 receptors and serotonin 5-HT1A and 5-HT2A receptors. Studies using cariprazine as augmentation therapy in depression have shown it can induce nighttime arousal, typically considered an insomnia side effect. However, this effect might benefit patients with hypersomnia. Rates of insomnia in patients with depression treated with cariprazine are relatively low compared to the placebo group (61, 65). Exact numbers shown by researchers are 6.8% with a dose of 1-2mg/day (33), 7.4% of patients with a dose of 1.5-4.5mg/day (24), 10% of patients taking 3mg/day cariprazine (35) and 13.6% when taking 2-4.5mg/day of cariprazine (36, 66).

The results of Vieta and co-authors are very interesting, showing that 7.6 of patients on cariprazine in doses 1.5-4.5mg/day reported insomnia, but the group without cariprazine reported insomnia in 10% of patients (32). These results also leave space for further analysis since no one analyzed what type of insomnia was present in patients. The clearest assumption from these studies is a dose-response effect of cariprazine confirmed in studies on schizophrenia (67).

Central neurobiological mechanisms also regulate appetite and the amount of food consumed. The pathway that stimulates/increases appetite (which includes agouti-related peptide and neuropeptide Y) is in balance with the appetite-suppressing melanocortin pathway (that releases pro-opiomelanocortin) (68, 69). Additionally, hormones such as ghrelin and leptin, neurotransmitters, and other central mechanisms regulate these pathways (70). Some agents which are 5HT2c agonists (like lorcaserin) are been registered for weight loss (71). Cariprazine is a low-potency 5HT2C antagonist, possibly leading to mild weight gain. However, it does not significantly interfere with appetite regulation pathways and is not associated with metabolic side effects, such as weight gain, commonly seen with other second-generation antipsychotics (27).

Results of published research on cariprazine augmentation in MDD confirm that body weight changes were not meaningfully different than placebo (34) or only a slight increase in mean body weight (1 to 2kg) vs placebo was observed (66). For instance, Riesenberg reported that mean changes from baseline in weight were <1kg in all groups: placebo 0.20kg; cariprazine 1.5mg/day-0.68kg; cariprazine 3mg/day – 0.66kg (35). Durgam and his team also reveal similar data: in all groups, the percentage of patients with >7% increase from baseline in body weight was low: placebo 1.9%, 1-2mg/day 1.5%, 2-4.5mg/day 3.3% (36). Although a majority of reports outline less than 5% of patients on cariprazine and antidepressant therapy had a weight increase of more than 7% and in similar distribution compared to placebos (32, 61) some authors communicated that a more significant number of patients had at least 7% increase in body weight with cariprazine 1.0–2.0 mg/day (15.1%) than in placebo (3.7%) or cariprazine 0.1–0.3 mg/day (1.3%) (33). It’s also important to emphasize that few researchers reported weight decreases in psychotic patients taking cariprazine (65).

Regarding nausea, some authors report lower rates of this adverse event in patients treated with cariprazine compared to the placebo group (32). Other researchers report low dose-dependent rates: for the placebo group (subjects taking only antidepressant-AD), nausea was described in 3.6% to 4.9% of patients, in those who were taking AD plus cariprazine 0.1-0.3mg/day 5.3% of patients had nausea, further ones on 1-2mg/day of cariprazine plus AD therapy reported nausea in 5.6% to 7% (33–36, 66). Patients on 3mg/day cariprazine plus AD had nausea around 6.4%, and the group on the highest dose of cariprazine up to 4.5mg/day revealed nausea in up to 12.8% (33–36, 66).

### Fatigue

Fatigue, a pervasive feeling of constant exhaustion and a subjective lack of physical and/or mental energy, interferes with daily and desired activities and is one of the most common symptoms of depression (72). Neurobiological studies have identified several key structures involved in the neural network regulation of fatigue, including the ascending arousal system, sleep executive control areas, reward-related regions, and the suprachiasmatic nucleus (73). Selective serotonin reuptake inhibitors were often mentioned as drugs that can reduce fatigue in depression (74). Still, this area is not fully investigated due to the complex interplay of many central neurotransmitters, cytokines, and chromones that are involved in the process of mentioned areas (75).

Atypical antipsychotics remain promising due to their effects on many of the aforementioned brain regions. Most studies exploring cariprazine in MDD have interpreted fatigue as a side effect. However, this interpretation is questionable, as fatigue could also be a manifestation of MDD itself. Depression can present in different eco-phenotypes, leading to varying levels of fatigue among patients. Recent research findings support this claim, showing that fatigue prevalence ranges from 0.5% to 1.9%, 2.5%, and up to 4.1% (32–34, 36). In that research, where patients were treated with AD and cariprazine augmentation, fatigue was observed in 1.3% of subjects who were taking cariprazine in a dose of 0.1-0.3mg/day, 6.6%-6.8% of patients on 1-2mg/day of cariprazine and 4.1%-9.5% in patients on 2-4.5mg/day of cariprazine (32–34, 36). Fatigue appeared to be dose-dependent, but data on potential fatigue reduction in other patients are lacking. Although fatigue has not been fully explored in cariprazine treatment until now, some research on depression in bipolar disorder highlights that cariprazine was effective at certain doses of 1.5-3mg/day (75).

### Psychomotor activity and anxiety

Psychomotor activity is often altered in major depressive disorder and abnormalities are manifested as psychomotor agitation and retardation (76). Research showed that low levels of striatal dopamine in the left hemisphere were correlated with psychomotor retardation in depressed patients (77). Similarly, Meyer and coauthors demonstrated in their work that insufficient dopaminergic transmission positively correlates with psychomotor retardation, primarily through the enhancement of D2 receptor and dopamine transporter binding potential (78, 79). On the other hand, agitation typically arises when the prefrontal cortex (PFC) loses its inhibitory control over the amygdala, GABA activity is low, and perception within the striatum and amygdala is heightened. Atypical antipsychotics address agitation and anxiety through several mechanisms: acting as α1 receptor antagonists, decreasing subcortical adrenoreceptor binding, and modulating subcortical serotonergic pathways (80).

Considering the neurobiology and cariprazine’s pharmacological profile, it seems unlikely that cariprazine is suitable for the treatment of anxiety disorders. Some authors have reported adverse events associated with its use, such as insomnia and anxiety in patients, which further discourage its application for these conditions (81). One randomized, double-blind, and placebo-controlled study on depressed patients at the endpoint after 8 weeks of cariprazine treatment in dosage 1.5-4.5mg/day did not find a significant reduction in anxiety by the Hamilton Anxiety Rating Scale (HAMA) scale, and anxiety was higher in cariprazine group (68). In research by Vieta and coauthors where doses of 1.5-4.5mg/day cariprazine where added to the AD therapy in patients with MDD, anxiety was registered in 1.7% of patients, and it was treated as an adverse effect (32). Considering the various manifestations of depression, cariprazine is likely not efficient and remains insufficiently investigated in this domain.

Concerning psychomotor activity, cariprazine could have varying effects, either reducing or inducing activity, through its agonism of 5-HT1A and 5-HT2A receptors and partial agonism of D2/D3 receptors. Unfortunately, there is insufficient evidence-based information to provide clear clinical data regarding its impact on agitation or psychomotor retardation.

Csehi and the team conducted a detailed analysis of agitation and psychomotor retardation in all case studies involving cariprazine. They found that one author reported psychomotor retardation in three psychotic patients, with one case becoming so severe that the patient became fully bedridden. Agitation and restlessness were commonly reported symptoms in patients before cariprazine treatment. However, most patients experienced a reduction in these symptoms in response to cariprazine. Conversely, in three cases, cariprazine induced agitation, which led to the discontinuation of the treatment (65).

Further analysis of this domain is necessary to understand better the effects of cariprazine on psychomotor activity, including its potential to either reduce or induce agitation and psychomotor retardation.

### Cognition

Cognitive impairments in MDD affect learning and memory, executive functioning, processing speed, attention, and concentration. The neurobiology of cognitive deficits in MDD involves disturbances in the structure, function, and interconnectivity of brain circuits and networks related to cognitive control and functioning (82). Circuits involving the orbitofrontal cortex (OFC), dorsolateral prefrontal cortex (DLPFC), anterior cingulate cortex, and key structures such as the hippocampus and amygdala are particularly relevant in MDD. These areas, coupled with well-established monoamine abnormalities, contribute to impaired cellular signaling and neurocircuit deficits associated with cognitive impairments in MDD (83, 84). The structure, function, and neurochemical composition of frontotemporal and fronto-subcortical circuits have also been implicated in the origination of cognitive symptoms in MDD. These circuits play a crucial role in developing cognitive deficits associated with the disorder (85, 86).

Cariprazine impacts many of the aforementioned neurochemical pathways, such as dopaminergic, serotonergic, and noradrenergic systems, making it worthy of detailed exploration concerning its effects on the cognitive domain in depression. A significant number of neurobiological studies on D3 receptors and cognition preceded the use of cariprazine in psychiatric syndromes. Papp and colleagues highlighted that the role of D3 receptors in modulating cognition emerged from studies showing patterns of D3 receptor expression in the brain, studies in D3 receptor knockout mice, and the upregulation of D3 receptors by antidepressants (52). Further, Nakajima and his team in a systematic review of the literature published several important findings regarding D3 receptors and cognition: D3 receptors are associated with cognitive functioning in both healthy individuals and those with neuropsychiatric disorders; D3 receptor agonism appears to enhance while D3 receptor blockade seems to impair cognitive function, including memory, attention, learning, processing speed, social recognition and executive function independent of age; and D3 receptor agonism may exert their pro-cognitive effect by enhancing the release of acetylcholine in the prefrontal cortex, disinhibiting the activity of dopamine neurons projecting to the nucleus accumbens or prefrontal cortex, or activating CREB signaling in the hippocampus (87). Those findings led to the investigation of the hypothesis that cariprazine has positive effects on cognition (88).

Animal model studies assumed that significant occupancy of the D3 receptor may contribute to cariprazine efficiency to diminish cognitive impairments (89–91). For instance, in an animal model of phencyclidine-induced cognitive impairment, cariprazine pretreatment significantly diminished phencyclidine-triggered cognitive deficits in social interaction/recognition and recognition memory, spatial working memory, and attention-set-shifting (91). Although we have data from animal models, none of the studies investigating cariprazine effects in Patients with MDD have separately analyzed cognitive impairments such as attention alteration, lack of concentration, and changes in the form and content of thinking. On the other hand, the hypothesis that cariprazine may be effective in improving cognitive dysfunction is supported by promising results from studies on bipolar depression (53).

## Discussion

Major depressive disorder is one of the most prevalent psychiatric disorders and a significant comorbidity factor with other psychiatric and somatic illnesses. Given the diverse manifestations of MDD, it has been proposed that symptoms be viewed through clusters to enable a more personalized treatment approach. Several authors have suggested their own clusters, typically ranging from four to five symptom clusters (20).

Despite the availability of various antidepressant treatments, approximately two-thirds of patients do not respond to initial antidepressant therapy administered at adequate doses and duration (23). This inadequate response necessitates the implementation of various strategies to address this issue. One of the most common strategies is the augmentation of antidepressants with atypical antipsychotics. Augmentation strategies aim to enhance the effectiveness of antidepressants by targeting different neurotransmitter systems and alleviating treatment-resistant symptoms (92). Cariprazine is a notable example of an atypical antipsychotic used for this purpose. It is one of the five antipsychotics approved by the FDA for the adjunctive treatment of TRD. Recent randomized, placebo-controlled studies that used cariprazine for MDD treatment have validated its role as an adjunct for MDD, though results vary concerning its advantage over placebo (33, 35–37).

Considering the domains of pleasure, interest, and motivation, and their reduction or loss as central symptoms of MDD, it is clear that cariprazine, through its agonism at D3 receptors, can potentially address these issues by enhancing reward-related behaviors. Early evidence—including animal studies and preliminary human research—suggests cariprazine’s potential to improve these symptoms by influencing effort-based choice behavior and attenuating anhedonia. This is particularly significant given the role of the mesolimbic system, which is rich in D3 receptors and integral to the brain’s reward circuitry. By targeting these receptors, cariprazine may help restore the motivation and interest that are often diminished in individuals with MDD. Additionally, the influence on effort-based choice behavior indicates a potential to improve the capacity for decision-making and the pursuit of rewarding activities, which are crucial for daily functioning and overall quality of life (48, 50, 53).

Depressive mood pathogenesis is multifaceted, encompassing an array of brain regions’ key neurotransmitter and cariprazine by targeting D2 and D3 dopamine receptors, as well as 5HT1A, 5HT2A, and alpha 1B receptors, offers a novel approach to treatment, as evidenced by its modulation of neurotransmission and neuron activity in animal studies. Following the same line of investigation, a considerable amount of research and meta-analyses suggest that the introduction of cariprazine as an augmentation therapy in the treatment of depression significantly reduces depressive symptoms compared to placebo. For bipolar depression with or without mixed features, cariprazine demonstrated clinical efficacy. Considering cariprazine’s potential to increase agitation, this might indicate a better therapeutic response in inhibited forms of depression.

Research on cariprazine’s impact on suicidality in depression indicates generally low rates of suicidal ideation, between 5-8%, with no significant increase in suicidal behavior reported during treatment. A study by Vieta et al. (32) observed that out of 442 patients treated with cariprazine as an adjunct therapy for MDD, only one case of treatment-related suicidal ideation was reported, suggesting that cariprazine may reduce or at least not exacerbate suicidality in patients with depression.

Moving on to the next cluster concerning sleep and appetite, the diverse symptoms of depression, especially in these areas, reflect the complexity of underlying neural mechanisms. The role of 5HT2A/2C receptors in promoting wakefulness underscores the nuanced approach required in treating depression-related sleep issues. Cariprazine’s role as a serotonin receptor partial agonist and its antagonist activity on H1 receptors suggest it may promote wakefulness, potentially leading to insomnia as a side effect in depression treatment. Interestingly, some studies suggest its insomnia side effects might be less prevalent than in control groups, indicating a dose-response relationship that warrants further exploration in the context of both depression and schizophrenia.

Studies on cariprazine’s effects on body weight in the treatment of MDD show minimal differences from placebo, with some studies noting slight increases in weight (1 to 2kg) (33). Data indicates a low percentage of patients experiencing a significant weight increase (>7% from baseline) across various doses, which is comparable to placebo effects (33). However, a subset of patients reported more notable weight gain at lower doses (33). At the same time, a few studies observed weight decreases in psychotic patients on cariprazine (65), suggesting a complex dose-response relationship concerning weight changes.

Research on cariprazine’s impact on MDD-related fatigue often categorizes it as a side effect, with incidence rates ranging from 0.5% to 9.5% across various doses. The observed dose-dependent increase in fatigue suggests a nuanced understanding of its role in MDD treatment is needed. Despite limited data on fatigue reduction, studies in the context of bipolar disorder suggest cariprazine’s potential effectiveness at doses of 1.5-3mg/day (84).

Cariprazine is unlikely to be effective for treating anxiety disorders, as studies have shown it may increase anxiety and insomnia in patients, and a randomized study found no significant reduction in anxiety symptoms (32, 34). Its impact on psychomotor activity is inconsistent, with reports of both reduction and induction of agitation and psychomotor retardation in patients. More research is needed to clarify cariprazine’s effects on anxiety and psychomotor symptoms.

Cognitive impairments in MDD span a spectrum of deficits, including learning and memory, executive functioning, processing speed, attention, and concentration. Studies on D3 receptors and cognition have indicated that D3 receptor blockade enhances cognitive function, whereas D3 receptor agonism impairs it (93). Animal model studies suggest that cariprazine’s efficacy in mitigating cognitive impairments may be attributed to its significant occupancy of D3 receptors (91). However, clinical investigations into cariprazine’s effects in Patients with MDD have yet to comprehensively analyze cognition impairments specifically. Nonetheless, promising findings from studies on bipolar depression suggest that cariprazine may hold promise for ameliorating cognitive dysfunction (53). Further research is crucial to elucidate the potential of cariprazine in addressing cognitive deficits associated with MDD.

The narrative review we conducted may have several limitations. The absence of quantitative analysis, unlike systematic reviews or meta-analyses, limits our ability to assess effect sizes or statistical significance. Variability in study quality can affect the reliability of our conclusions. The review’s scope, limited to studies published up to January 2024 and in English, may exclude recent and non-English research, impacting comprehensiveness. Our subjective interpretation may introduce bias. Lastly, the review’s narrow focus might not encompass all relevant aspects of cariprazine’s efficacy and safety. These limitations should be considered when interpreting the findings.

## Conclusions

Cariprazine, a dopamine partial agonist, exhibits a unique pharmacological profile with distinct effects on D3 receptors, 5HT1A, 5HT2A, and alpha 1B receptors, making it pharmacologically distinct from other antipsychotics. Its specific actions on D3 and 5-HT1A receptors suggest that cariprazine could be a promising candidate for the treatment of MDD across various domains. Research on animal models indicates its potential benefits for addressing impaired motivation, anhedonia, and cognitive impairments.

However, there is currently insufficient information regarding cariprazine’s effects on agitation, psychomotor retardation, and anxiety. Based on its pharmacological profile, cariprazine could mitigate or exacerbate these symptoms. Patients with MDD who might benefit from cariprazine augmentation include those with anhedonia, cognitive problems, and probably with hypersomnia.

By examining the efficacy of cariprazine across different symptom domains, we aim to identify patterns that could guide more personalized and effective treatment strategies for depression. Further research is essential to elucidate the role of cariprazine as an adjunctive therapy for adults with major depressive disorder who have an inadequate response to antidepressant monotherapy.
